# Non-Functionalized Fullerenes and Endofullerenes in Aqueous Dispersions as Superoxide Scavengers

**DOI:** 10.3390/molecules25112506

**Published:** 2020-05-28

**Authors:** Ivan V. Mikheev, Madina M. Sozarukova, Elena V. Proskurnina, Ivan E. Kareev, Mikhail A. Proskurnin

**Affiliations:** 1 Department of Chemistry, Lomonosov Moscow State University, 119991 Moscow, Russia; mikheev.ivan@gmail.com; 2 Kurnakov Institute of General and Inorganic Chemistry, Russian Academy of Sciences, 117901 Moscow, Russia; s_madinam@bk.ru; 3 Research Centre for Medical Genetics, 115522 Moscow, Russia; proskurnina@gmail.com; 4 Institute of Problems of Chemical Physics of the Russian Academy of Sciences, Chernogolovka, 142432 Moscow Region, Russia; kareev@icp.ac.ru

**Keywords:** Fullerene aqueous dispersions, non-functionalized (pristine) fullerenes, nanozymes, chemiluminescence, SOD-mimic activity, reactive oxygen species

## Abstract

Endohedral metal fullerene are potential nanopharmaceuticals for MRI; thus, it is important to study their effect on reactive oxygen species (ROS) homeostasis. Superoxide anion radical is one of the key ROS. The reactivity of aqueous dispersions of pristine (non-functionalized) fullerenes and Gd@C_82_ endofullerene have been studied with respect to superoxide in the xanthine/xanthine oxidase chemiluminescence system. It was found that C_60_ and C_70_ in aqueous dispersions react with superoxide as scavengers by a similar mechanism; differences in activity are determined by cluster parameters, primarily the concentration of available, acting molecules at the surface. Gd endofullerene is characterized by a significantly (one-and-a-half to two orders of magnitude) higher reactivity with respect to C_60_ and C_70_ and is likely to exhibit nanozyme (SOD-mimic) properties, which can be accounted for by the nonuniform distribution of electron density of the fullerene cage due to the presence of the endohedral atom; however, in the cell model, Gd@C_82_ showed the lowest activity compared to C_60_ and C_70_, which can be accounted for by its higher affinity for the lipid phase.

## 1. Introduction

Reactive oxygen species (ROS) play an important part in regulating many biological processes in living systems [1,2]. ROS are closely related to cell proliferation, growth and death, redox signaling, immune function, inflammation, carcinogenesis, ageing, and degenerative processes [3,4]. Among ROS, hydrogen peroxide and superoxide anion radical (SAR) are the two key redox signaling agents generated under the control of growth factors and cytokines by more than 40 enzymes, prominently including NADPH oxidases and the mitochondrial electron-transport chain [5]. Produced in proper amounts, superoxide is a normal and relevant metabolite serving as a signaling molecule. But when overproduced, this radical can initiate lipid peroxidation, protein oxidation, and DNA damage, hence leading to cell dysfunction and death by apoptosis or necrosis [6]. 

For SAR metabolization, a single enzyme family, superoxide dismutases (SOD), is responsible. They catalyze the dismutation of two SARs to hydrogen peroxide and molecular oxygen as 2OO^•–^ + 2H;^+^ = H;_2_O;_2_ + O;_2_. SODs are metalloproteins with nickel, iron/manganese, or zinc/copper active centers [7]. As SAR is involved in the inflammation development, SOD mimics can possess anti-inflammatory effects [8]. In fact, protective and beneficial roles of SOD enzymes in many diseases have been shown, both preclinically and clinically [9]. 

The main drawbacks of these natural species are their large size, which hinders cell permeability, their short circulating half-life, antigenicity, and expense. Thus, several low-molecular SOD mimics have been developed to overcome some of these drawbacks [10]. To-day, many comprehensive reviews sum up the knowledge on the properties of compounds with SOD-mimic activity [11,12]. Among these compounds, promising are artificial nanozymes. Recently, they have become increasingly attractive as therapeutic agents because they can be imbued with many target properties and are stable in vivo. One of nanozyme candidates are fullerenes [13], first of all, their functionalized species because structural and electronic parameters of their cages provide various targeted chemical grafting resulting in biochemically active water-soluble derivatives [14]. Such functionalized fullerenes are efficient peroxidase and SOD mimics [15,16,17,18]. 

However, nowadays, the research is focused on non-functionalized (pristine) fullerenes in aqueous dispersions as they are not involved in metabolic processes owing to their open surface without moieties. Pristine fullerenes show a protective antioxidant effect in ROS-dependent experimental models of cell damage [19,20,21,22,23,24,25]. Antioxidant effects of aqueous fullerene dispersions (AFD) have also been proved in vivo [26]. 

Another relevant fullerene nanozyme candidates are endohedral metallofullerenes (endofullerenes). Of particular interest are gadolinium endofullerenes as MRI agents [27,28] because of their efficiency and safety in comparison with commercially available drugs [29]. They can be also used in vivo for inhibiting tumor proliferation and initiating antioxidant defenses [30]. Biocompatibility and safety are even more important for endofullerenes because of their use in MRI and possible metal-releasing cage opening for functionalized species. However, antioxidant and SAR-scavenging properties of non-functionalized endofullerenes have been not thoroughly studied. 

Thus, the aim of this paper is to study SOD-mimic activity of aqueous dispersions of non-functionalized fullerenes and gadolinium endofullerenes in in vitro and cell models. 

## 2. Results

### 2.1. Preparation, Purification, and Characterization of Aqueous Fullerene Dispersions 

Two types of AFDs have been prepared for comparison sake: (1) By direct ultrasound sonication of pristine C_60_, C_70_, and Gd@C_82_ (*C_2v_*) in ultrapure water and (2) by ultrasound sonication with solvent replacement in ultrapure water. The direct sonication procedure without solubilizing agents was previously developed for the synthesis of fullerene derivatives [31]. Organic solvents (benzene or toluene) may also be used to prepare concentrated and stable solutions of C_60_, C_70_ [32], and Y@C_82_ (*C_2v_*) through solvent replacement [33]. However, after removing the solvents by ultrasonic-assisted evaporation, traces of harmful organic compounds may cause nanozyme inactivation [34]. 

We used an immersion ultrasound probe made of titanium. Despite the formation of titania nanoparticles during ultrasonication, this procedure produces efficiently dispersed nanoparticles [35]. According to ICP–AES, a prolonged ultrasound exposure of fullerene C_60_–water mixtures resulted in the concentration of total titanium as high as 3.50 ± 0.05 ppm. Cellulose syringe filters with a pore diameter of 0.45 and 0.22 μm were used to purify the dispersions from titanium. As a result, all the prepared samples contained less than 1 ppm titanium dioxide. 

The filtration also improves the optical parameters of AFDs (Figures S1 and S2 in Supplementary Materials), though the fullerene concentrations have been decreased to 50–60% of the initial (by UV/VIS absorbance [32] for C_60_ and C_70_ and ICP–AES for Gd@C_82_). ATR-FTIR spectra of pristine fullerenes and AFD C_60_ have been recorded (Figures S3 and S4 in Supplementary Materials). The absence of significant functionalization of fullerenes in AFDs has been proved by MALDI [33]. The details of the prepared samples are presented in Table 1.

### 2.2. Superoxide Scavenging by SOD 

**Figure 1:**
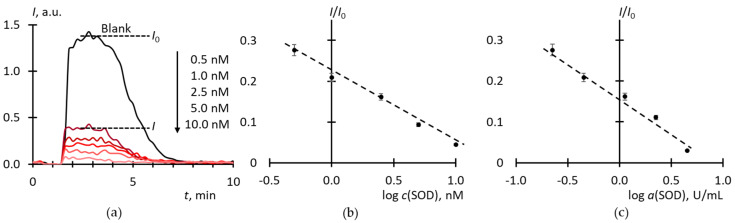
Oxidation of xanthine (10 μM) with xanthine oxidase (2.2 mU/mL) in the presence of lucigenin (10 μM) and various amounts of superoxide dismutases (SOD): (**a**) Source chemiluminograms, SOD concentrations are shown in the legend; (**b**) the dependence of the ratio of the stationary CL signal to the stationary signal control experiment *I*/*I*_0_ vs. log *c*_SOD_; (**c**) the same plot in *I*/*I*_0_ vs. log *a*_SOD_ coordinates; the total volume, 1000 mL; temperature, 37 °C.

The chemiluminescence (CL) system based on xanthine/xanthine oxidase (Xa/XO) and lucigenin is widely used for studying superoxide-scavenging potentialities of SOD mimics [36,37]. We have optimized the procedure to achieve reproducible steady-state levels of luminescence intensity and studied the effect of SOD as a reference enzyme (Figure 1). 

**Figure 2:**
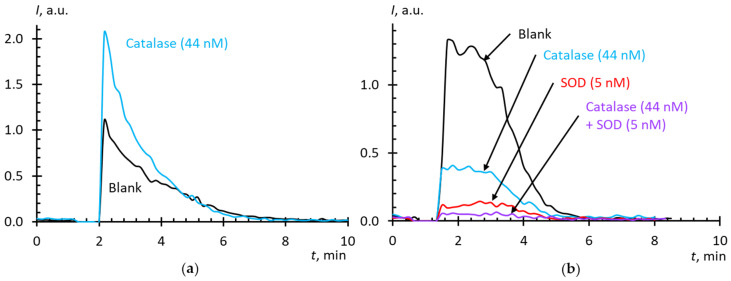
Chemiluminescence kinetics of xanthine (10 μM) oxidation with xanthine oxidase (2.2 mU/mL) in the presence of catalase (44 nM, 27.5 U/mL) and SOD (5 nM) and CL-enhancers: (**a**) luminol (20 μM); (**b**) lucigenin (10 μM); phosphate buffer solution (100 mM pH 7.4); the total volume, 1000 mL; temperature, 37 °C.

The area under a CL curve is proportional to the number of radicals formed, but in this case, we cannot use it as a measure of SOD antioxidant activity. This could be done if the chemiluminescence after the consumption of the antioxidant resource of SOD had returned to its previous level. However, the luminescence breaks off (the blank curve in Figure 1a), possibly due to the autoactivation of xanthine oxidase by the formed uric acid [38] or hydrogen peroxide [39], which, along with SAR, is a product of reactions catalyzed by xanthine oxidase [40,41]. Therefore, to build a calibration curve, we used the ratio of the stationary intensity to the intensity of the blank experiment as a function of log *c*_SOD_ (Figure 1b). To discriminate the nature of CL, experiments were carried out with the addition of catalase to Xa/XO with lucigenin and luminol (Figure 2). Lucigenin is a CL probe sensitive mainly to SAR, while luminol is sensible to both SAR and hydrogen peroxide [42,43].

### 2.3. Blank Experiments

**Figure 3:**
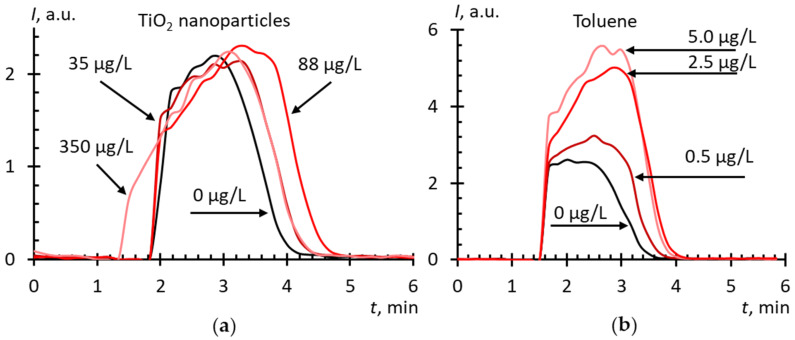
Chemiluminograms of aqueous dispersions of (**a**) TiO_2_ and (**b**) toluene in phosphate buffer solution (100 mM pH 7.4) + lucigenin (10 μM) + xanthine (10 μM) + xanthine oxidase (2.2 mU/mL); the total volume, 1.000 mL; temperature, 37 °C.

Titania nanoparticles were present in all AFDs because of the immersion probe technique, and traces of toluene were present in the case of solvent replacement. Blank experiments showed that the effect of titania nanoparticles can be neglected (Figure 3a). However, toluene showed pro-oxidant activity, which depended on the concentration (Figure 3b), which should be involved into the interpretation of experiments with solvent-replacement AFDs.

### 2.4. Superoxide Scavenging by Fullerenes in Aqueous Dispersions

**Figure 4:**
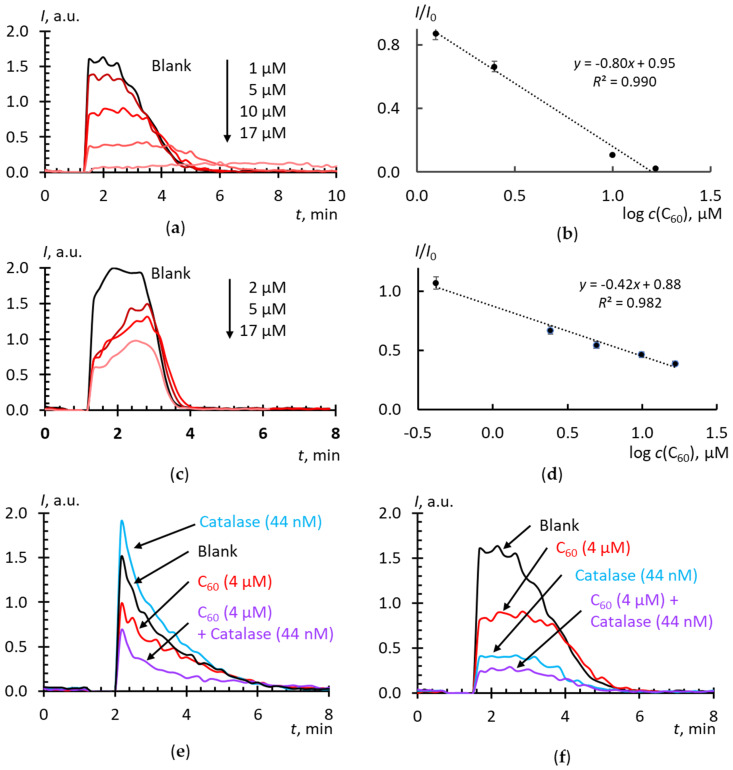
Effects of aqueous dispersions of C_60_ on the chemiluminescence kinetics of xanthine (10 μM) oxidation with xanthine oxidase (2.2 mU/mL): (**a**) C_60_ by direct dispergation, lucigenin-enhanced chemiluminescence (10 μM); (**b**) C_60_ by direct dispergation, calibration plot of *I*/*I*_0_ vs. lg(*c*); (**c**) C_60_ by solvent replacement, lucigenin-enhanced chemiluminescence (10 μM); (**d**) C_60_ by solvent replacement procedure, calibration plot of *I*/*I*_0_ vs. lg(*c*); (**e**) with catalase (44 nM, 27.5 U/mL) added, luminol-enhanced chemiluminescence, (20 μM); (**f**) with catalase (44 nM, 27.5 U/mL) added, lucigenin-enhanced chemiluminescence, (10 μM); C_60_ concentrations are given in plots, phosphate buffer solution (100 mM pH 7.4); the total volume, 1000 mL; temperature, 37 °C.

The results of studying the superoxide-scavenging properties of C_60_ AFDs by direct dispergation and with solvent replacement are shown in Figure 4a–d. The addition of C_60_ up to 20 μM; to Xa/XO decreases the luminescence that demonstrates their SAR scavenging ability. The equations of calibration curves and the concentration of half-suppression of the signal of the blank experiment (Table 2) show that: (a) C_60_ in AFD reacts with SAR about 10^5^ times less actively than SOD, and (b) an AFD by solvent replacement is a slightly more active scavenger of SAR than an AFD by direct dispergation, even despite of the prooxidant effect of toluene. Recalculation of the dependence from total fullerene concentration to the concentration of acting surface fullerene molecules does not lead to significant changes in the slopes (Table 2). 

**Figure 5:**
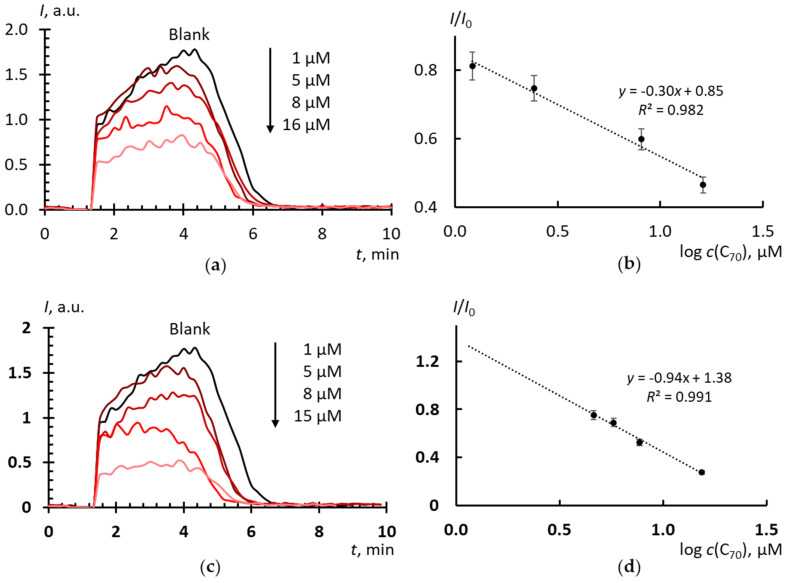
Effects of ADF C_70_ on the chemiluminescence kinetics of xanthine (10 μM) oxidation with xanthine oxidase (2.2 mU/mL): (**a**) C_70_ was prepared by direct dispergation procedure, lucigenin-enhanced chemiluminescence (10 μM); (**b**) C_70_ was prepared by direct dispergation procedure, calibration plot of *I*/*I*_0_ vs. log(c); (**c**) C_70_ was prepared by solvent replacement procedure, lucigenin-enhanced chemiluminescence (10 μM); (**d**) C_70_ was prepared by solvent replacement procedure, calibration plot of *I*/*I*_0_ vs. log(*c*); concentrations of C_70_ are given in plots, phosphate buffer solution (100 mM pH 7.4); the total volume, 1000 mL; temperature, 37 ℃.

For C_70_ (Figure 5), *c*_½_ values (Table 2) show that: (a) C_70_ in AFD reacts with SAR about 10^6^ times less actively than SOD and nearly five times less actively than C_60_ in AFD, (b) AFD by solvent replacement is a slightly more active SAR scavenger, as C_60_. The kinetics of suppressing the blank signal by C_70_ AFD (Figure 5a,c;) is close to that for C_60_ (Figure 4a,c), but not to the kinetics of SOD action (Figure 1a). 

**Figure 6:**
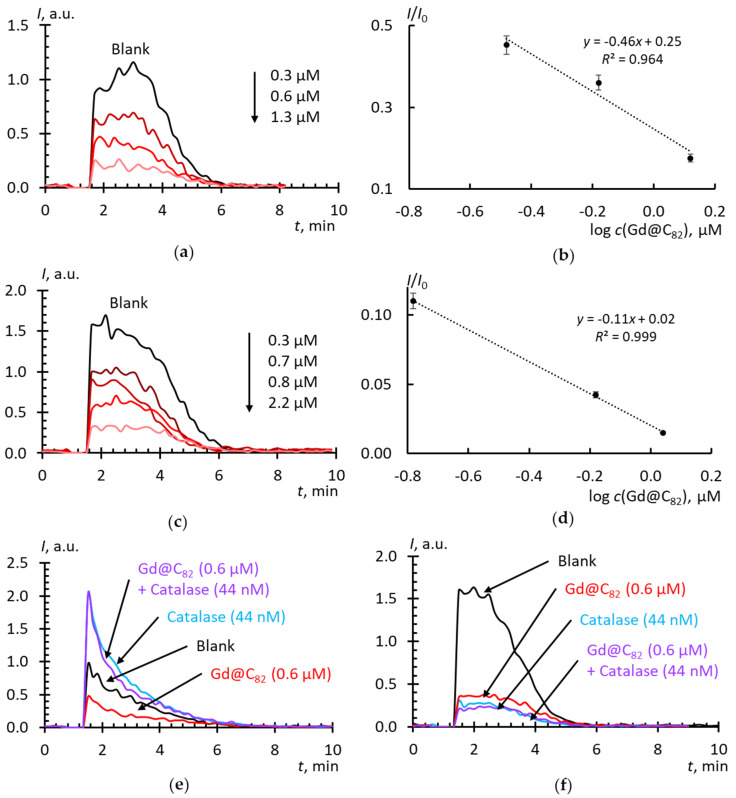
Effects of Gd@C_82_ on the chemiluminescence kinetics of xanthine (10 μM) oxidation with xanthine oxidase (2.2 mU/mL): (**a**) Gd@C_82_ by direct dispergation, lucigenin-enhanced chemiluminescence (10 μM); (**b**) Gd@C_82_ by direct dispergation, calibration plot of *I*/*I*_0_ vs. lg(*c*); (**c**) Gd@C_82_ by solvent replacement, lucigenin-enhanced chemiluminescence (10 μM); (**d**) Gd@C_82_ by solvent replacement procedure, calibration plot of *I*/*I*_0_ vs. lg(*c*); (**e**) with catalase (44 nM, 27.5 U/mL) added, lucigenin-enhanced chemiluminescence, (10 μM), (**f**) with catalase (44 nM, 27.5 U/mL) added, lucigenin-enhanced chemiluminescence, (20 μM); concentrations of Gd@C_82_ are given in plots, phosphate buffer solution (100 mM pH 7.4); the total volume, 1000 mL; temperature, 37 ℃.

Experiments in the Xa/XO + lucigenin system for Gd@C_82_ are shown in Figure 6a–d. In the system with lucigenin and luminol, the effect of the addition of catalase (Figure 6e,f) was tested. From the *c*_½_ values (Table 2) it follows that (a) Gd@C_82_ reacts with SAR approximately 10^4^ times less actively than SOD, and this fullerene is about 10–15 times more active than C_60_ and 50 times than C_70_ AFDs; (b) as in the previous two cases, the fullerene obtained by solvent replacement is a slightly more active SAR scavenger. 

### 2.5. Superoxide Scavenging Potential of Aqueous Fullerene Dispersions in Cells

Considering the potential use of endofullerenes as contrasting agents for MRI, the next step is the study of superoxide-scavenging properties in cells. It is believed that it is the SOD-like effect of nanopharmaceuticals that can reduce their cyto- and genotoxicity. Such studies may include the study of cytotoxicity using the MTT test, visualization of the permeation of nanoparticles into cells and their distribution based on intrinsic fluorescence by confocal microscopy, visualization of intracellular ROS using, for example, H2DCFH-DA (7’-dichloro-dihydrofluorescein diacetate). Here, we conducted preliminary studies of the SAR-scavenger potential of the fullerenes on human fetal lung fibroblasts using the lucigenin + NADH chemiluminescent system as a model for the generation of intracellular superoxide by cytochrome b5 reductase [44,45]. 

**Figure 7:**
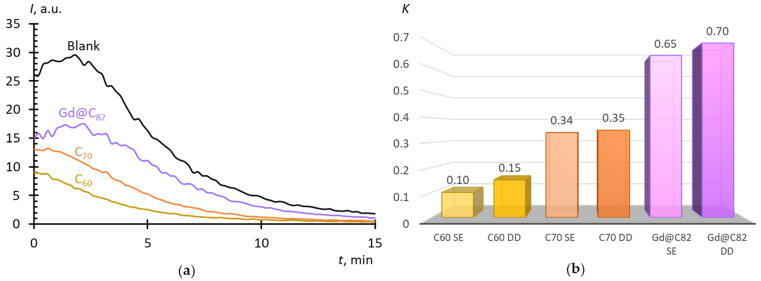
(**a**) Chemiluminograms of aqueous dispersions of fullerenes (17 μM, direct dispergation) in a Krebs-Ringer solution + human fibroblasts (1.0 × 10^6^ cells in mL) + lucigenin (0.4 mM)+NADH (0.8 mM); (**b**) Suppression constants *K* = *S*/*S*_0_ for AFDs; SE is solvent replacement, DD is direct dispergation; the total volume, 1000 mL; temperature, 37 ℃.

Figure 7a shows NADH-stimulated lucigenin-enhanced chemiluminescence kinetics in the presence of the AFDs. From the chemiluminograms, the area under the curve for 20 min was determined, and the degree of suppression of luminescence related to the blank was calculated (*K* = *S*/*S*_0_). The lower the K value, the higher the SAR-scavenging potential of the substance. 

## 3. Discussion

Thus, the results obtained can be summed up as follows.

1. The AFDs prepared by solvent replacement have a slightly higher antioxidant activity than those prepared by direct dispersion.
2. With both methods of AFD preparation, fullerenes can be arranged in the row Gd@C_82_ > C_60_ > C_70_ with respect to the ability to scavenge SAR; and C_60_ and C_70_ differ in the mechanism of interaction with SAR from SOD, which allows them to be rather considered superoxide scavengers, in contrast to Gd@C_82_, which, presumably, is a SOD mimic.
3. With respect to the intracellular SAR, the activity of fullerenes decreases in the row C_60_ > C_70_> Gd@C_82_.

This requires considering all the factors that may affect the reactivity of fullerenes.

### 3.1. Preparation Procedure

As AFDs consist of fullerene clusters of ca. 100 nm in diameter (Table 1), not the whole molecular fullerene concentration should be taken into account. In fact, a more correct treatment of the concentration parameters is the cluster concentration or the concentration of fullerene molecules at the surface, an acting part of the fullerenes should be considered (Table 1).

The influence of the preparation method can be explained by the size of the obtained particles, the direct dispersion method is reported to result in larger particles [46]. The average cluster size for AFD C_60_ prepared by solvent-replacement was 277 nm, for direct dispergation, 357 nm [47]. In this study, even after filtering through a 220 nm filter, the average particle size in the direct dispergation AFD was larger than the particle size obtained by solvent replacement (Table 1). A suspension of larger nanoparticles would provide a smaller total surface area for interaction and provide less reactive sites for ROS, thus reducing the efficiency of scavenging reactive species. The influence of the shape of fullerene molecules and their symmetry on the nature of cluster formation in AFD was discussed using the examples of C_60_ and C_70_ [48]. C_70_ is characterized by the formation of larger clusters. The primary factor in the aggregation and, apparently, the nature of the formed cluster of molecules can be the dielectric constant (*ε*) of solvent molecules, so for the aggregation of C_70_ it was shown that *ε* = 27 ÷ 31 are needed, for the aggregation of C_60_ the required range of *ε* = 12 ÷ 14. Here, we observed identical values of the sizes of colloidal fullerene particles, and comparable absolute values of electrokinetic potentials.

Another explanation for differences in antioxidant activity of fullerenes may be the sorption of benzoic acid on the cluster surface at the process of solvent replacement. Toluene is dispersed in this process, and due to the oxidizing atmosphere, high ultrasound power, and water sonolysis [49] with the formation of the OH· radical, toluene is oxidized to benzoic acid [50]. Benzoic acid itself has a pronounced antimicrobial effect [51], and its hydroxylated derivatives (2-hydroxybenzoic acid, etc.,) have an antioxidant effect beyond the phenol-like structure [52]. However, the complete elucidation of the composition of such impurity components was beyond the scope of this work.

Finally, when using the solvent replacement technique, even after cleaning, a solvent may remain in the system, which helps to form clusters with solvent molecules included [53,54]. Solvent molecules form a film-like solvate structures with water and/or toluene. In the case of the solvent replacement procedure, clusters with a nanoporous structure (~120 nm) can form [46,54], which are formed in toluene and then diffuse into the aqueous phase. Previously, we showed [55] that porous structures are characteristic for C_60_ and C_70_ clusters in AFDs by solvent replacement and can be isolated as solids. Presumably, the formed pores in fullerene clusters can serve as additional sites that increase the reactivity of fullerenes. There is data on the concentration dependence of AFD, the cluster sizes of which decrease with dilution [54]; however, we did not observe similar behavior for C_60_, C_70_, and Gd@C_82_.

### 3.2. Chemiluminescence System Based on Xanthine/Xanthine Oxidase

It is interesting that the catalase addition to the Xa/XO + luminol system increased the CL intensity (Figure 2a), which indicates the formation of extra ROS. This compound is unlikely to be singlet oxygen [56], but rather a hydroxyl radical formed by the Haber–Weiss reaction in the interaction of superoxide and hydrogen peroxide [57,58,59,60] or by the Fenton reaction with iron present in catalase samples [61]. It is possible that Fe(III) is formed during the oxidative degradation of catalase, as described in the study of the interaction of tert-butyl hydroperoxide with catalase [62]. There is evidence that SAR itself can have a damaging effect on catalase [63]. The confirmation of this hypothesis is in the study [64], where a sharp increase in lipid peroxidation by xanthine oxidase is observed upon adding catalase to the system, and this effect was inhibited by not only SOD, but iron chelators as well. The authors believe that the reduction of Fe(III) with superoxide to Fe(II) served as an effective trigger for lipid peroxidation. 

Another possible explanation is the side peroxidase effect of catalase described in a few studies [65] when catalase catalyzes the oxidation of organic substrates in the presence of hydrogen peroxide. The “hydroxyl radical generation theory” was also proposed, according to which mammalian catalases generate hydroxyl radicals apart from their main catalytic reaction [66,67]. This effect depends on the concentration of hydrogen peroxide. At low concentrations of hydrogen peroxide, it is possible that the reaction mechanism switches, and ROS are formed instead of water and oxygen. However, such a prooxidant effect of catalase in the Xa/XO + luminol system was not shown [68], the authors observed the expected decrease in CL. Such a difference with our results may be related to the reagent ratios. At approximately the same concentration of luminol (20 μm in this study and 25 μm in [65], 1:1.25), we used 5–10-fold lower xanthine concentrations (10 μm compared to 50–500 μm) and 18-fold lower xanthine oxidase activity (2.2 U/mL compared to 40 U/mL).

In our blank experiments for luminol (20 mM) + hydrogen peroxide (20 mM) + catalase (44 nM, 27.5 U/mL), an expected decrease in the CL signal was observed (the data not shown), which is consistent with the published data [69], but the interpretation of the catalase effect in a complex system for the generation of hydrogen peroxide by xanthine in the presence of xanthine oxidase requires further studies.

The addition of catalase to the Xa/XO + lucigenin system led to the suppression of luminescence, which either indicates the presence of H_2_O_2_, to which lucigenin is also sensitive [70], or can be explained by the reaction of Fe(III) with SAR. It is highly likely that the effect of catalase on both luminol-dependent and lucigenin-dependent CL is due to the formed iron.

Thus, despite the widespread use of the Xa/XO as a model, its reaction mechanism is extremely complex, which does not make it possible to simulate the kinetics and thus suggest a reaction scheme. Hence, we limited ourselves to comparing the properties of AFDs toward SAR relative to SOD as quantitative estimates and hypotheses on the mechanism of action; the proof of the latter may be a goal of separate studies involving other methods.

### 3.3. Activity Mechanisms 

The study of the antioxidant properties of fullerenes is given special attention, since it is believed that they determine cytotoxicity, and non-functionalized fullerenes are more toxic to cells because of their lower antioxidant properties. The molecular mechanisms behind the antioxidant reactions of fullerenes with SAR remain controversial. Previously, the direct reaction between radical species and the highly conjugated double bond system of C_60_ was considered as the main mechanism. The authors [71] even referred to C_60_ as a free-radical “sponge” because the large polarizability of fullerenes [72] enables to attach radicals. Fullerenes show this row of electron affinity: C_60_ < C_70_ < Gd@C_82_; the values are 2.683(8), 2.765(10), and 3.3(1), respectively [73]. 

Figure 4a, c show that C_60_ acts differently than SOD, as the plateau in this case reaches the blank level (compare with Figure 1a). Presumably, this means that substances that inhibits xanthine oxidase and hydrogen peroxide do not form. It could be assumed that C_60_ neutralizes hydrogen peroxide; however, our experiments in the CL system of hydrogen peroxide and luminol showed that C_60_ is inert with respect to peroxide or exhibits very weak peroxidase properties (data not shown). We also did not find any previous evidence of the catalase-like properties of pristine fullerenes. Therefore, it can be assumed that these differences are associated exactly with the different mechanism of reactions of SOD and C_60_ with superoxide. Probably, in this case, C_60_ should be considered not as a SOD mimic acting according to the catalytic mechanism, but rather a superoxide scavenger. Finally, Figure 4e reveals that C_60_ inhibits a luminol-dependent CL, probably because of a decrease in SAR concentration, and its reaction with superoxide is faster than the reaction of superoxide with luminol. Noteworthy is that catalase in the presence of C_60_ does not exhibit a pro-oxidant effect. It can be assumed that C_60_, because of its antioxidant properties and rapid reaction kinetics, protects catalase from damage by SAR.

From Figure 1, Figure 4, and Figure 5 it follows that, most likely, the mechanism of interaction with superoxide for C_60_ and C_70_ is similar, and differences in activity are associated with the electron density in these molecules.

The comparison of Figure 1a and Fiugre 6a,c shows that the mechanism of the reaction of Gd@C_82_ with SAR is similar to the mechanism of SOD action and different from two other fullerenes. In both cases, in the presence of an antioxidant, the luminescence ends earlier, and its termination is characterized by a slope depending on the antioxidant concentration. However, Gd@C_82_ does not protect catalase from oxidative damage: the level of luminol-dependent CL in Figure 6e is the same for catalase and catalase + Gd@C_82_ cases. Perhaps this is due to a lower reaction rate of Gd@C_82_. 

Presumably, the superoxide anion reacts with Gd@C_82_ through a catalytic mechanism. This hypothesis is implicitly supported by the unchanged absorption and fluorescence spectra of Gd@C_82_ AFD in the course of the reaction with superoxide (the data not shown). To correctly substantiate this hypothesis, it is necessary either to carry out mathematical modeling, which, as discussed above, is not possible because of the complexity of the reaction mechanism in the Xa/XO + lucigenin system, or, along with determining the concentration of SAR from lucigenin CL, to determine the amount of hydrogen peroxide formed as a result of dismutation. This cannot be quantified using peroxidase, since xanthine inhibits this enzyme [74], or by spectrophotometry because of the interfering effect of endofullerene. A way out could be the use of an electrochemical generation of a superoxide anion radical [75]. Therefore, we consider that the results obtained should be developed in further studies.

Much more studies have been devoted to fullerene derivatives, which consider being more efficient antioxidants in contrast to pristine fullerenes. For example, tris-malonic acid derivatives (C3) could remove the superoxide radical approximately 100-fold slower than SOD that was comparable to the range of values reported for several manganese-containing SOD mimic compounds, and C3 acted through the catalytic dismutation of superoxide [18,76,77]. Antioxidant properties of carboxyfullerenes depended on charge, size, shape, and hydrophobicity [78] as well as on the dipole moment [79]. The SOD activity of different carboxyfullerenes was also dependent on their reduction potentials; the higher the reduction potential, the higher the SOD dismutation activity [80]. Another example is hydrophilic carbon clusters that are oxidized carbon nanoparticles with a high affinity for electrons, which react with superoxide through a two-electron process with the formation of H_2_O_2_ as well as poly(ethylene glycolated) hydrophilic carbon clusters [81,82]. The authors used chemiluminescence techniques to prove that nanoparticles of fullerenols Gd@C_82_(OH)_22_ and C_60_(OH)_22_, as well as malonate-adduct C_60_(C(COOH)_2_)_2_ were more efficient scavengers of SARs than water-soluble underivatized C_60_ [22]. They paid special attention that the derivatization of the fullerene cage with carboxyl and hydroxyl groups resulted in a drastic decrease in the ROS-induced cytotoxicity. They also demonstrated that Gd-endohedral fullerenes were much stronger ROS scavenger than hollow fullerenes [22]. 

Our results are consistent with this: Gd@C_82_ turned out to be an order of magnitude more effective antioxidant against superoxide than C_60_ and C_70_. The radical-scavenging abilities of fullerenes may be attributed to significant electron affinity: 2.7 eV for C_60_ and 3.14 eV for C_82_ [83,84]. The insertion of Gd into a C_82_ cage increases the electron affinity to 3.3 eV [84]. Moreover, large polarizability of fullerenes facilitates the attachment of radicals to their surface [85]. Differences in electron affinities may contribute to the relative scavenging efficiencies Gd@C_82_ > C_60_ and C_70_. 

The catalytic activity of endofullerenes can be accounted for by the asymmetric position of the endohedral Gd with respect to the carbon skeleton, and at least two carbon atoms of fullerene are more strongly bound to the endohedral atom by non-covalent interactions. According to [86], the electrons of the cage are bound more strongly because of their electrostatic interaction with the endohedral atom, which facilitates making Gd@C_82_ a stronger electron acceptor [86]. More negative redox potentials by cyclic voltammetry [86,87,88] indicate that endohedral metal fullerenes have a better reduction ability compared to C_60_ or C_70_, which is indirectly confirmed by electron affinity energies.

In addition, the introduction of a metal atom or other entity into the fullerene cage leads to the appearance of “extra” electrons in the π-electronic subsystem, the number of which depends on the valency or charge state of the embedded entity [89,90]. This explains the different behavior of endofullerenes compared to non-endohedral fullerenes. The most stable configuration for Gd@C_82_ is when the gadolinium atom is adjacent to the double bond of the carbon skeleton (the mechanism of the in-plane inter-Gd@C_82_ interaction on the electronic features, which would be helpful for qualitatively addressing the origin of the scanning tunneling spectroscopy states) [91].

### 3.4. Intracellular SAR-Scavenging Activity 

With respect to intracellular superoxide, fullerenes can be arranged in a row C_60_ > C_70_ > Gd@C_82_, and C_60_ (*K* = 0.15) was a 2–3-fold more efficient intracellular superoxide scavenger than C_70_ (*K* = 0.35) (Figure 7b). This is consistent with the Xa/XO molecular model. However, the scavenging potential of Gd@C_82_ (*K* = 0.62) was two-fold lower than that of C_70_ and five times lower than C_60_. Thus, unlike the molecular model, Gd@C_82_ turned out to be the weakest antioxidant, which can be accounted for by a larger nanoparticle size. However, when evaluating intracellular activity, the permeability of substances through the membrane should be taken into account. Thus, such a row can be explained by higher lipid solubility (high hydrophobicity) that leads to accumulation in the cell membrane in the initial period of incubation. Here, the incubation time was 15 min that was enough for permeation of C_60_ and C_70_ into the cells but not for Gd@C_82_. In the future, it is necessary to study the dynamics of the effect and vary the incubation time, but here it is clear that Gd@C_82_ will exhibit antioxidant properties with respect to the intracellular SAR.

In the case of unmodified fullerenes (especially for endofullerenes) there is no existing data on the strength of hydrophobic interactions. However, some conclusions may be made by the comparison of modified fullerenes. First, it was found that negatively charged C_60_ aggregates accumulate on deposited lipid bilayers consisting of cationic lipid head groups to a greater extent than on bilayers consisting of only zwitterionic lipid head groups under the same conditions [92]. Thus, it may be concluded that fullerene clusters interacted only with lipid head groups and did not penetrate lipid hydrocarbon chains. Also, the penetration time of C_70_–*γ*-cyclodextrin from AFD into the membrane for HeLa cells is ca. 10 min, whereas it was almost not observed for more stable C_60_–*γ*-cyclodextrin complex [93]. As well, permeability constants for AFDs at pH 7.4 in a model biological membrane (L/mg × kg lipid), are 3.30 for C_60_ and 3.08 for C_60_(OH)_24_ [94]. These values suggest that the more hydrophilic fullerenol is less bound because of the electrostatic repulsion forces. A stronger hydrophobic and electrostatic interaction promotes more stable adsorption of Gd@C_82_(OH)_22_ compared to other fullerenes [95]. Gd@C_82_(OH)_22_ is relatively more hydrophobic compared to C_60_(OH)_24_ because of the larger number of carbon atoms and fewer hydroxyl groups [95]. Meanwhile, the negatively charged endofullerene framework Gd@C_82_ because of electron transfer from the central atom [89] promotes electrostatic interaction with collagen, especially at the N-terminal nitrogen atom. 

Thus, our data provide the basis for in-depth studies on cell models with a focus on studying the permeation and distribution of these substances and their intracellular antioxidant activity.

## 4. Materials and Methods 

### 4.1. Chemicals

Pristine C_60_ and C_70_ (>99.5%) fullerenes were purchased from Limited Liability Scientific and Production Company NeoTechProduct (St. Petersburg, Russia). The soot containing the Gd@C_2n_ endohedral metal fullerenes (total content of Gd atoms up to 4 wt.% checked by ICP-AES, and the value of total Gd was recalculated to the general formula of the molecule, Gd@C_82_) has been synthesized by the evaporation of the composite graphite electrodes compounded by gadolinium in the electric arc reactor as we previously described elsewhere [96]. Standard reference materials and quality control standards of required elements with certified values (Inorganic Ventures^TM^, Christiansburg, VA, USA) were used to conduct ICP-AES measurements. A 20 ppm (in 5 wt. % HNO_3_) scandium solution was used as an internal standard. Ultrapure water Milli-Q® Type (Merck, Darmstadt, Germany) was applied during the research (TOC <3 ppb). 

Phosphate buffer solutions (PBS) with 100 mM and pH 7.4 and 8.6 were prepared by dissolving a weighed portion of KH_2_PO_4_ (Sigma-Aldrich, St. Louis, MO, USA) in 1000 L of ultrapure water, followed by adjusting to the desired pH value using granular KOH (Sigma-Aldrich, USA). Solutions of xanthine (3,7-dihydropurine-2,6-dione, Sigma-Aldrich, USA) and a selective superoxide anion-radical of a chemiluminescent probe, lucigenin (10-methyl-9-(10-methylacridin-10-ium-9-yl)acridin-10-ium dinitrate, Sigma-Aldrich, USA), with working concentrations of 1 mM, was prepared by dissolving weighed portions in PBS with pHs of 8.6 and 7.4, respectively. A xanthine oxidase working solution (Sigma-Aldrich, USA, X1875-25UN) with an activity of 0.11 U/mL was obtained by diluting the initial PBS suspension (pH 7.4). Before luminescence measurements, the working solution of the enzyme was kept for 15 min at laboratory temperature. The catalytic activity of the one enzyme unit converts 1.0 μmol of xanthine to uric acid in 1 min at 25 °C and pH 7.5. A stock solution of NADH (10 mM) (Sigma-Aldrich, USA) was prepared by dissolving a weighed portion of the substance in distilled water. Catalase from bovine liver, 2000–5000 U/mg protein was purchased from Sigma-Aldrich. A weighed portion of 0.0011 g was dissolved in 1000 mL of distilled water that corresponds to a 2750 U/mL activity. A 44 nM solution of catalase corresponds to 27.5 U/mL final activity.

### 4.2. Sample Preparation and Characterization

AFDs was prepared by direct or solvent-replacement methods with a commercially available off-the-shelf ultrasound probe with a timer MEF93.T (LLC MELFIZ-ul’trazvuk, Moscow, Russia). Ultrasonic tip (surface areas 0.63 ± 0.02 cm^2^) and electrical-power mode (0.6 kW) were used. Ultrasound tips were made of titanium alloys, grade TM3 (ISO 28401:2010). 

#### 4.2.1. Preparation of Aqueous Fullerene Dispersions by Direct Ultrasound Probe Sonication

The weighted fullerene portion of ca. 0.05 g, 10 mL of toluene, and 50 mL of ultrapure water were subsequently added to a conical flask (250 mL). The solution was exposed to ultrasonic treatment for 12 h with every 60 min pause for 30 min. The prepared solution was filtered through a 0.45 μm cellulose filter and diluted to the mark with 50 mL of ultrapure water.

#### 4.2.2. Preparation of Aqueous Fullerene Dispersions by Solvent-Replacement Ultrasound Probe Sonication

The weighted fullerene portion of ca. 0.05 g, 10 mL of neat toluene, and 50 mL of ultrapure water were subsequently added to a conical flask (250 mL). The solution was exposed to ultrasonic treatment for 12 h with every 60 min pause for 30 min. The prepared solution was filtered through a 0.45 μm cellulose filter and diluted to the mark with 50 mL of ultrapure water. StrataX solid-phase extraction cartridges (Phenomenex, Torrance, CA, USA) were used for purified dispersion and eliminate organic compounds. 

#### 4.2.3. Characterization

The colloidal characteristics of aqueous fullerene dispersions (particle size distribution and ζ-potential) were determined by dynamic light scattering using a ZetaSizer Nano ZS (Malvern PANalytical, Malvern, UK) operating at 25 °C, the angle of backscattering 173° according to ISO 22412:2017. An Agilent 720 ICP-OES spectrometer (Agilent Technologies Inc., Santa Clara, CA, USA) with an axial view was used for elemental analysis. 

### 4.3. Chemiluminescence Experiments 

A 12-channel Lum-1200 chemiluminometer (DISoft, Moscow, Russia) was used for chemiluminometry measurements. The chemiluminometer provides the detection of visible light within a range from 300 to 700 nm. No light filters were used in our experiments. Signal processing and data handling were performed with PowerGraph 3.3 Professional software (DISoft, Moscow, Russia). 

#### 4.3.1. Chemiluminescence Analysis of Superoxide Scavenging Potential

Superoxide scavenging potential was assessed by chemiluminometry with the Xa/XO model system in the presence of lucigenin as a selective enhancer for SAR. Aliquots of xanthine (25 μM), lucigenin (25 μM), and the analyzed sample were added to a cuvette with the PBS (100 mM, pH 7.4). The background luminescence was recorded for 30–60 s, then an aliquot of xanthine oxidase (*a* = 5.5 mU/mL) was added. The total volume of the system was 1000 mL. The samples were analyzed in triplicates. As an analytical signal, the ratio of the of the stationary intensity to the intensity of the control experiment *I*/*I*_0_ was used. Experiments were performed at 37 °C. 

To confirm that AFDs did not inhibit xanthine oxidase activity, we measured both uric acid and O_2_ consumption during the reaction. Oxygen content was assessed using a Clark type electrode DTKP-02 (Ekoniks-Ekspert, Moscow, Russia). Uric acid was determined electrochemically [97]. The formation of uric acid, i.e., xanthine oxidase activity, was not inhibited by any AFDs (data not shown).

#### 4.3.2. NADH-Stimulated Lucigenin-Enhanced Chemiluminescence of Human Fibroblasts

Human fetal lung fibroblasts (the fourth cell passage) were provided by the Research Centre for Medical Genetics. Cells were seeded at 17 × 10^4^ per mL in DMEM (Dulbecco’s Modified Eagle Medium) (Paneco, Moscow, Russia) with 10% fetal calf serum (PAA, Vienna, Austria), 50 U/mL penicillin, 50 μg/mL streptomycin, and 10 μg/mL gentamycin, and cultured at 37 °C for 24 h. After adding the fullerenes to the medium, the cells were incubated for 15 min.

The cells were placed into a cuvette with Krebs-Ringer buffer solution and lucigenin (0.4 mM). Chemiluminescence was recorded at 37 °C; for 2 min, then NADH solution were added (0.8 mM) and the chemiluminescence was recorded for 15 min. From the chemiluminograms, the area under the curve for 20 min was determined, and the suppression of luminescence relative to blank was calculated (*K* = *S*/*S*_0_).

### 4.4. Cluster Parameters in Aqueous Fullerene Dispersions

We assumed a spherical structure of the clusters and the fact that fullerenes form a face-centered cubic lattice. The outer average diameter of the molecules: for C_60_ 6.5 Å, [98] for the c-axis C_70_ 7.96 Å [99] (values 7.12 Å along the a and b-axes were not used), and Gd@C_82_ 14.30 Å [100]. The cluster concentration of fullerenes in AFD *ć* (Table 1) was calculated as: *ć* = (1/*k*)(*d*/*D*)^3^(*cN*_A_/1000*M*), where *D* is the average diameter of the fullerene cluster, nm, *d* is the diameter of the fullerene molecule C_60_, C_70_, or Gd@C_82_, nm, *c;* is the *w/v* concentration of fullerene in the sample (mg/L), *N_A_* is Avogadro’s number, *M* is the molar mass of the fullerene (g/mol), and *k* is the packing coefficient of the face-centered cubic lattice of 0.7404. We also estimated: (*i*) The number of molecules in one cluster *n*_c_ = (*D*/*d*)^3^*k*; (*ii*) the number of molecules on the surface of the cluster, taking into account that only a half of the surface of the molecule works, and the rest is inside the cluster by the formula *n*_c_,_surf_=2(*D*/*d*)^2^; (*iii*) the fraction of active (surface) fullerenes in cluster is the ratio of the number of molecules on the surface of the cluster to their total number in the cluster (fraction of the working surface of the cluster) as *η=n*_c_,_surf_/*n*_c_ × 100%; and (*iv*) acting (surface) fullerene concentration as *ć*, act=*ć*/(*N_A_n*_c_,_surf_). The calculated parameters are summed up in Table 1. 

## 5. Conclusions

Based on the results of studying SOD scavenging activity, the following conclusions can be made: (1) C_60_ and C_70_ aqueous dispersions react with superoxide as scavengers by a similar mechanism, differences in their activity are determined by cluster parameters, primarily the concentration of active (acting) molecules at the surface; (2) Gd endofullerene is characterized by a significantly (one and a half to two orders of magnitude) higher reactivity compared to non-endohedral fullerenes and is likely to exhibit SOD-mimic catalytic properties, which can be explained by the nonuniform distribution of electron density of the fullerene cage due to the presence of an endohedral atom; (3) slightly higher antioxidant activity of aqueous fullerene dispersions by the method of solvent replacement is most likely due to the effect of benzoic acid adsorbed at the fullerene clusters; (4) in the cell model, Gd endofullerene showed the lowest activity compared to C_60_ and C_70_, which may be accounted for by a higher affinity for the lipid phase preventing its appearance within the cell. Still, these studies may be considered as a proof-of-concept. The continuation of these studies may be helpful in: (1) studying antioxidant potentialities of aqueous fullerene dispersions in relation to other biochemical reference systems (lipoperoxidases or phospholipoperoxidases), including computer-based simulation of the kinetics mechanism and (2) studying fullerenes in aqueous dispersions as regulators of cellular ROS homeostasis, for example, cytotoxicity, permeation into cells and their distribution, and visualization of intracellular ROS in the presence of fullerenes. Also, it could be mentioned that the shown ability of Gd endofullerenes as intracellular superoxide scavengers would make them promising cytoprotective MRI contrasting agents.

## Figures and Tables

**Table 1:** Concentrations (*c;*), size, zeta-potential (**ζ**), and cluster parameters of aqueous fullerene dispersions including cluster concentrations (*ć*), the number of molecules in a cluster (*n*_c_), the number of molecules at the cluster surface *(n*_c_, _surf_), the fraction of active (surface) fullerenes in cluster (*η*), and acting (surface) fullerene concentration *(ć*, act)

Fullerene	Preparation Technique	*c;*, mM	Particle Size after a 0.22 μm Filter, nm	ζ-Potential, mV	*ć* × 10^–13^, Clusters/L	*ć*, pM	*n*_c_ × 10^–6^	*n*_c_,_surf_ × 10^–4^	*η*,%	*ć*, act, μM
C_60_	Direct sonication	0.083^1^	110 ± 5	–28.4 ± 0.2	1.40	23.2	3.6	5.7	1.60	1.3
C_60_	Solvent-replacement sonication	0.090^1^	100 ± 3	–29.0 ± 0.3	2.01	33.5	2.7	4.7	1.76	1.6
C_70_	Direct sonication	0.081^1^	113 ± 2	−29.5 ± 0.3	2.30	38.2	2.1	4.0	1.90	1.5
C_70_	Solvent-replacement sonication	0.077^1^	111 ± 3	–30.9 ± 0.3	2.32	38.5	2.0	3.9	1.94	1.5
Gd@C_82_	Direct sonication of solid Gd@C_82_-enriched sample	0.022^2^	95 ± 5	–32.3 ± 0.3	6.07	100.1	0.20	0.88	4.07	0.89
Gd@C_82_	Solvent-replacement sonication of toluene HPLC-grade Gd@C_82_ solution	0.011^2^	90 ± 2	–25.2 ± 0.3	3.43	59.9	0.18	0.79	4.29	0.45

^1^ measured by UV/vis spectroscopy. ^2^ measured by ICP–AES.

**Table 2:** Antioxidant capacity of aqueous fullerene dispersions (AFD) C_60_, C_70_, Gd@C_82_, and SOD (*n* = 3, *P* = 0.95).

Sample	Calibration Functions *I*/*I*_0_ vs.*c* (or *a* for SOD)	Calibration Functions *I*/*I*_0_ vs. Active Cluster Concentration (*ć*, act)	Concentration of Semi-Suppression of Reference (Blank) CL (*c*_1/2_)	Rel. to SOD Efficiency, × 10^6^
SOD	*I*/*I*_0_ = (–0.18 ± 0.01) × *a*(U/mL) + (0.16 ± 0.03), *r* = 0.9820	—	0.03 ± 0.005 nM	—
AFD C_60_ (direct dispergation)	*I*/*I*_0_ = (–0.80 ± 0.09) × *c* (µM) + (0.96 ± 0.06), *r* = 0.9980	*I*/*I*_0_ = (–0.60 ± 0.06) × *c* (µM) – (0.36 ± 0.06), *r* = 0.9952	4.0 ± 0.1 µM	7.5
AFD C_60_ (solvent replacement)	*I*/*I*_0_ = (–0.42 ± 0.03) × *c* (µM) + (0.87 ± 0.5), *r* = 0.9960	*I*/*I*_0_ = (–0.27 ± 0.03) × *c* (µM) + (0.70 ± 0.22), *r* = 0.9771	2.0 ± 0.4 µM	15
AFD C_70_ (direct dispergation)	*I*/*I*_0_ = (–0.30 ± 0.02) × *c* (µM) + (0.85 ± 0.09), *r* = 0.9860	*I*/*I*_0_ = (–0.30 ± 0.02) × *c* (µM) + (0.8 ± 0.0), *r* = 0.9913	14.5 ± 2.1 µM	2
AFD C_70_ (solvent replacement)	*I*/*I*_0_ = (–0.94 ± 0.08) × *c* (µM) + (1.34 ± 0.3), *r* = 0.9980	*I*/*I*_0_ = (–0.94 ± 0.08) × *c* (µM) + (0.23 ± 0.07), *r* = 0.9913	9.0 ± 0.7 µM	3
Gd@C_82_ (direct dispergation)	*I*/*I*_0_ = (–0.46 ± 0.3) × *c* (µM) + (0.25 ± 0.05), *r* = 0.9640	*I*/*I*_0_ = (–0.46 ± 0.3) × *c* (µM) + (0.25 ± 0.05), *r* = 0.9818	0.28 ± 0.05 µM	100
Gd@C_82_ (solvent replacement)	*I*/*I*_0_ = (–0.12 ± 0.01) × *c* (µM) + (0.02 ± 0.002), *r* = 0.9980	/*I*_0_ = (–0.12 ± 0.02) × *c* (µM) + (0.13 ± 0.04), *r* = 0.9998	0.07 ± 0.005 µM	400

## Supplementary Materials

The following are available online, Figures S1–S2: UV/vis spectra of different types of aqueous dispersions (filtered and not-filtered); Figures S3–S4: FTIR–ATR spectra of the pristine fullerenes and their aqueous dispersions.
