# Supplementary material for: Non-Functionalized Fullerenes and Endofullerenes in Aqueous Dispersions as Superoxide Scavengers
Source: Molecules. 2020 May 28;25(11):2506. doi: 10.3390/molecules25112506 (PMC7321068; doi:10.3390/molecules25112506)
Supplement: Supplementary file 1 [file molecules-25-02506-s001.pdf]

Supplementary Materials

of

Non-functionalized fullerenes and endofullerenes in aqueous dispersions as superoxide scavengers

by

Ivan V. Mikheev [1], Madina M. Sozarukova [2], Elena V. Proskurnina [3], Ivan E. Kareev [4], and Mikhail A. Proskurnin[1]

[1] *Department of Chemistry, Lomonosov Moscow State University, Moscow, 119991 Russia*

[2] *Kurnakov Institute of General and Inorganic Chemistry, Russian Academy of Sciences, Moscow, 117901 Russia*

[3] *Research Centre for Medical Genetics, Moscow, 115522 Russia*

[4] *Institute of Problems of Chemical Physics of the Russian Academy of Sciences 142432 Chernogolovka, Moscow Region, Russia.*

E-mail: mikheev.ivan@gmail.com, proskurnin@gmail.com

Table of Contents

1. Ultraviolet-visible absorbance measurements....2

2. Fourier-transform infrared spectroscopy....4

3. References of supplementary materials....6

1. Ultraviolet-visible absorbance measurements

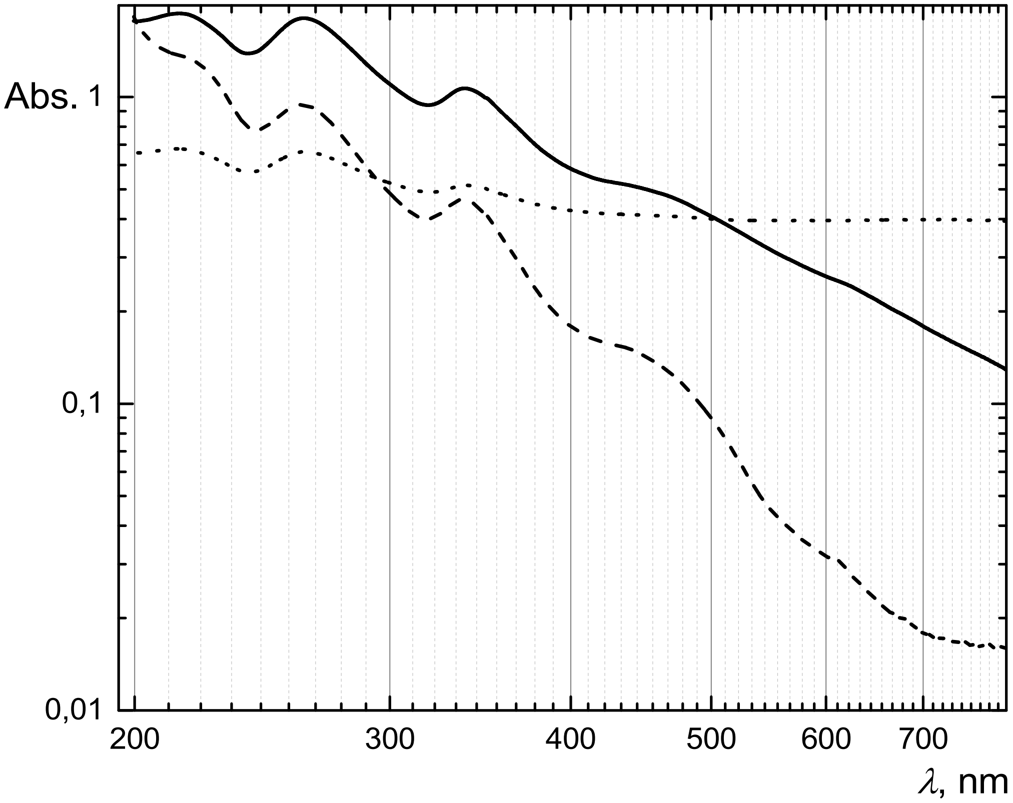

Figure S1. Absorbance spectra in the log-log coordinates of aqueous fullerene dispersions of C_{60} in the range 190–800 nm:

(i) native (dotted line) with a significant scattering component (pathlength 0.2 cm, 37.9 ± 0.5 ppm);

(ii) filtered through 0.22 µm (dashed line) with a lower scattering component (pathlength 1.0 cm, 15.7 ± 0.6 ppm);

(iii) sample after sedimentation during 24h (solid line) with an appropriate scattering component (pathlength 1.0 cm, 6.8 ± 0.7 ppm);

scanning rate 40 nm/min, data interval 0.067 nm, integration time in each point 1.0 s.

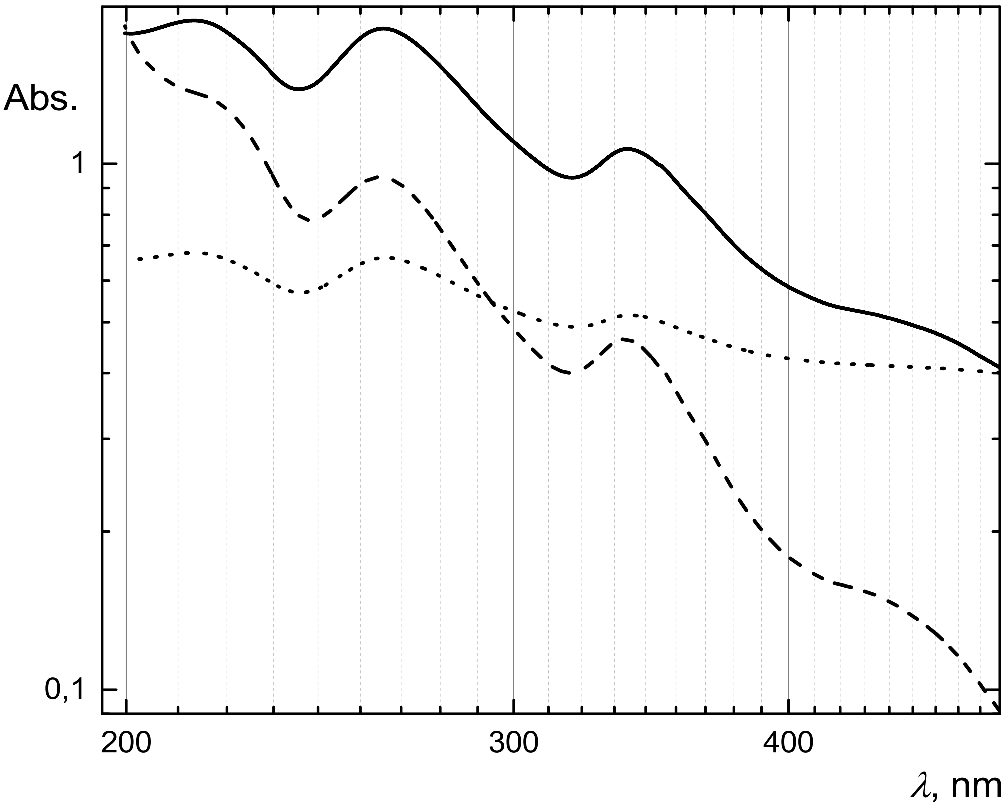

Figure S2. Absorbance spectra in the log–log coordinates of aqueous fullerene dispersions of C_{60} in the range 190–500 nm:

(iv) native (dotted line) with a significant scattering component (pathlength 0.2 cm, 37.9 ± 0.5 ppm);

(v) filtered through 0.22 μm (dashed line) with a lower scattering component (pathlength 1.0 cm, 15.7 ± 0.6 ppm);

(vi) sample after sedimentation during 24h (solid line) with an appropriate scattering component (pathlength 1.0 cm, 6.8 ± 0.7 ppm);

scanning rate 40 nm/min, data interval 0.067 nm, integration time in each point 1.0 s.

Filtering or samples sedimentation improves their spectral characteristics for all aqueous dispersions (C_{60}, C_{70} and Gd@C_{82}). The scattering of the filtering samples (through 0.22 μm) is decreased, and the peaks (absorbance maxima) become narrower. As well, we isolated a narrow-sized fraction of fullerene nanoparticles with an average cluster diameter *ca.* 121 ± 5 nm. As well, absorbance maxima at 261 and 338 nm have coincided, but still, sample filtering allowed us to observe additional peaks at 447 nm (mid absorption band in a range 400–550 nm) and 607 nm. According to [1] the absorbance spectra coincided with a slight red shift in this work.

2. Fourier-transform infrared spectroscopy

The FTIR spectroscopy was applied to clarify the nature and the state of titanium and silicon particles. According to ICP–AES titanium and silicon was observed after an ultrasound probe sonication of fullerene–water systems. Owing to the possible generation of free radicals by sonolysis of water, it was supposed to check the metal or nonmetal oxide formation.

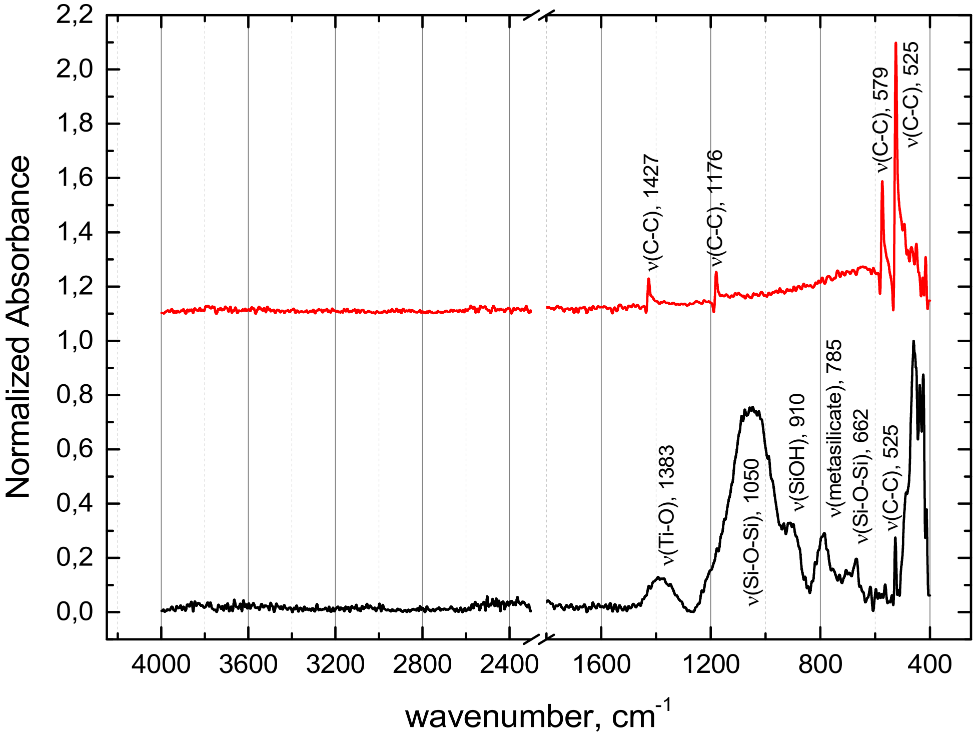

Figure S3. FTIR-Absorbance spectra in the range 4000–400 cm^{-1} of pristine C_{60} (red line), concentrated AFD C_{60} (black line) by centrifugation, scans — 64, baseline scans — 64 data interval — 1 cm^{-1}.

The FTIR-ATR spectra of pristine C_{60} include all C–C bands [2], as for AFD C_{60} obtained by a direct probe sonication of water–fullerene suspension, additional signals are observed. Figure S3 shows of AFD C_{60} FTIR spectra. The bands of SiO_2, TiO_2 NPs are present. In Figure S3 (line) the FTIR spectrum of TiO_2 NPs band clearly shows the signal at 1383 cm^{-1} related to Ti-O modes [3-5]; band at 1050 cm^{-1} correspond to silicon oxide, Si–OH or Si–O–Si modes [6,7]. For more detail, the figure with a narrower range is given (Figure S4)

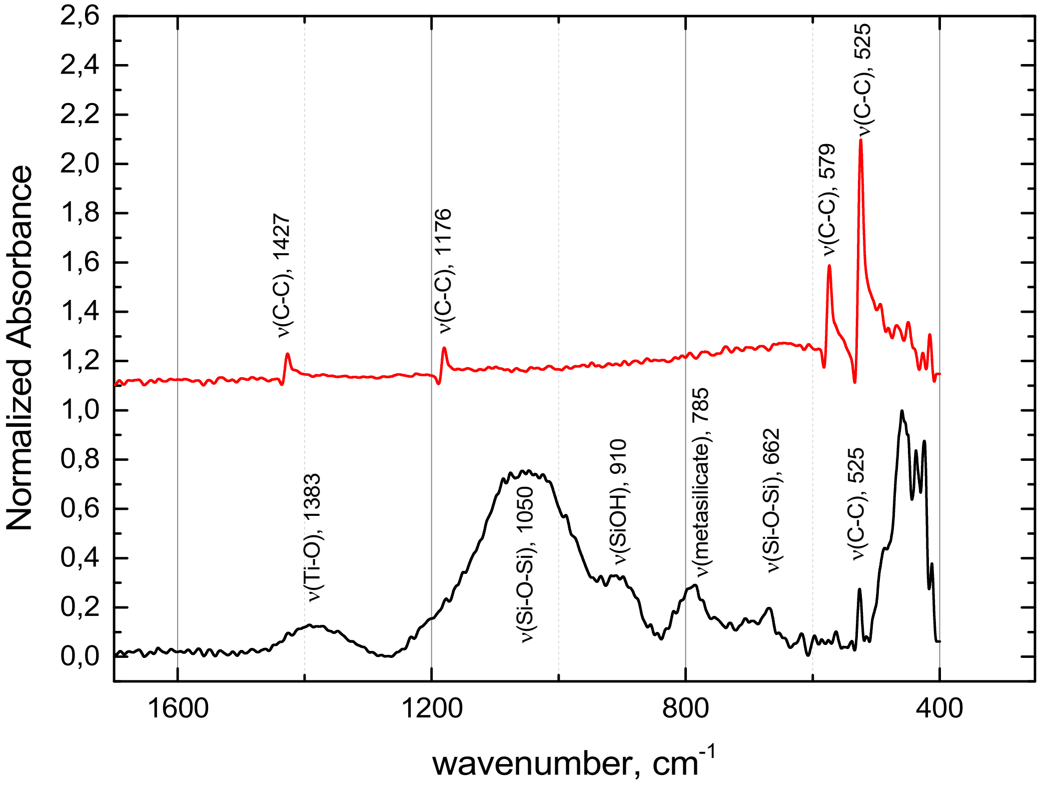

Figure S4. FTIR-Absorbance spectra in the range 1650–400 cm^{-1} of pristine C_{60} (red line), concentrated AFD C_{60} (black line) by centrifugation, scans — 64, baseline scans — 64 data interval — 1 cm^{-1}.

3. References in supplementary materials

1. Brant, J.A.; Labille, J.; Bottero, J.-Y.; Wiesner, M.R. Characterizing the Impact of Preparation Method on Fullerene Cluster Structure and Chemistry. *Langmuir* **2006**, *22*, 3878-3885, doi:10.1021/la053293o.
2. Prylutskyy, Y.I.; Petrenko, V.I.; Ivankov, O.I.; Kyzyma, O.A.; Bulavin, L.A.; Litsis, O.O.; Evstigneev, M.P.; Cherepanov, V.V.; Naumovets, A.G.; Ritter, U. On the Origin of C60 Fullerene Solubility in Aqueous Solution. *Langmuir* **2014**, *30*, 3967-3970, doi:10.1021/la404976k.
3. Leon, A.; Reuquen, P.; Garin, C.; Segura, R.; Vargas, P.; Zapata, P.; Orihuela, P.A. FTIR and Raman Characterization of TiO2 Nanoparticles Coated with Polyethylene Glycol as Carrier for 2-Methoxyestradiol. *Appl. Sci.-Basel* **2017**, *7*, 9, doi:10.3390/app7010049.
4. Mugundan, S.; Rajamannan, B.; Viruthagiri, G.; Shanmugam, N.; Gobi, R.; Praveen, P. Synthesis and characterization of undoped and cobalt-doped TiO2 nanoparticles via sol–gel technique. *Applied Nanoscience* **2015**, *5*, 449-456, doi:10.1007/s13204-014-0337-y.
5. Abazović, N.D.; Čomor, M.I.; Dramićanin, M.D.; Jovanović, D.J.; Ahrenkiel, S.P.; Nedeljković, J.M. Photoluminescence of anatase and rutile TiO2 particles. *The Journal of Physical Chemistry B* **2006**, *110*, 25366-25370.
6. Davidson, G.; Dillon, K.B. *Spectroscopic Properties of Inorganic and Organometallic Compounds*; Royal Society of Chemistry: 2007.
7. Oh, T.; Choi, C.K. Comparison between SiOC Thin Films Fabricated by Using Plasma Enhance Chemical Vapor Deposition and SiO2 Thin Films by Using Fourier Transform Infrared Spectroscopy. *J. Korean Phys. Soc.* **2010**, *56*, 1150-1155, doi:10.3938/jkps.56.1150.
